# Technologies for engineering repetitive DNA

**DOI:** 10.1002/qub2.70040

**Published:** 2026-04-21

**Authors:** Shuting Ma, Yali Cui, Yi Wu

**Affiliations:** ^1^ State Key Laboratory of Synthetic Biology Tianjin University Tianjin China; ^2^ Frontiers Science Center for Synthetic Biology and Key Laboratory of Systems Bioengineering (Ministry of Education) School of Synthetic Biology and Biomanufacturing Tianjin University Tianjin China; ^3^ Frontiers Research Institute for Synthetic Biology Tianjin University Tianjin China; ^4^ School of Chemical Engineering and Technology Tianjin University Tianjin China

**Keywords:** DNA assembly, genetic manipulation tools, genome synthesis, repetitive DNA engineering, synthetic biology

## Abstract

Repetitive DNA, a fundamental architectural element of genomes, is widespread across organisms and comprises about 54% of the human genome. With advances in long‐read sequencing and bioinformatics approaches, highly repetitive sequences can now be characterized in depth. Nevertheless, engineering repetitive DNA is mainly limited by repeat instability. Despite these challenges, advances have been reported in the construction of short tandem repeats and the large‐scale duplication of genes. This review provides a comprehensive overview of current strategies for constructing repetitive DNA, covering both in vitro approaches, including restriction endonuclease ligation, in vitro homologous recombination, and redundancy‐minimizing sequences, as well as in vivo methodologies in *Escherichia coli*, *Saccharomyces cerevisiae*, and mammalian cells. We further discuss applications of these methods for developing genetic manipulation tools, constructing models of repetitive DNA, and synthesizing biomaterials. Future progress in engineering repetitive DNA will further advance our capabilities in designing and manipulating complex biological systems and deepen our understanding of them.

## 1 INTRODUCTION

Repetitive DNA, defined as sequence motifs that recur throughout the genome, comprises about 54% of the human genome [1]. Large amounts of repetitive DNA are likely to be non‐functional, decaying remnants of transposable elements (TEs), which have been labeled “junk” or “selfish” DNA [2]. However, growing evidence suggests that repetitive sequences in both coding and noncoding regions play essential roles in evolution, regulation of gene expression, and the generation of genetic variation [3, 4, 5, 6]. Over 60 diseases have been identified as being caused by the expansion of simple repetitive DNA in the genome, with the majority affecting the nervous system, particularly trinucleotide repeat diseases such as Huntington’s disease, fragile X syndrome, several spinocerebellar ataxias, myotonic dystrophy, and Friedreich’s ataxia [7, 8].

Repetitive DNA is broadly categorized into two types: tandem repeats and interspersed repeats, according to the arrangement of repeat units [9]. Tandem repeats consist of head‐to‐tail arrays of repeat units. By repeat unit length, tandem repeats are classified into three categories: microsatellites (short tandem repeats, STRs), minisatellites (variable number tandem repeats, VNTRs), and satellite DNA [10]. STRs, defined as motifs of 1–6 bp, are enriched in regulatory regions, including enhancers, where they can modulate gene expression. Their instability leads to expression variability and underlies neurological repeat‐expansion disorders [11, 12]. VNTRs, defined as tandem arrays with repeat‐unit lengths of about 10–100 bp, have been shown to influence gene expression, protein function, and complex traits [13, 14, 15]. Satellite DNA is enriched in centromeric and pericentromeric heterochromatin and is often present at subtelomeric regions. It exhibits considerable variation in unit size across species, from very short simple satellites in insects to nucleosome‐length units in mammals. It contributes to chromosome segregation (kinetochore function), heterochromatin formation, and telomere‐associated processes [16, 17]. Interspersed repeats are repetitive DNA derived mainly from TEs, including Class I retrotransposons and Class II DNA transposons. Unlike tandem repeats, whose units are contiguous, interspersed repeats are dispersed throughout the genome and occur as nonadjacent copies [18, 19]. TEs can induce genomic instability through insertional mutations and chromosomal rearrangements. Conversely, they may also contribute to adaptive evolution by providing regulatory elements such as promoters and enhancers that influence gene expression [20, 21, 22, 23].

Advances in long‐read sequencing, combined with bioinformatics, enable the in‐depth characterization of highly repetitive genomic elements by resolving their repeat‐unit composition, higher‐order organization, and locus‐specific variation [24]. Nevertheless, the construction of repetitive DNA remains challenging, primarily stemming from structural instabilities caused by replication slippage during amplification, misassembly driven by high sequence homology, and genetic instability of constructs during serial passaging [25, 26]. To address these challenges, a suite of in vitro amplification‐based strategies has been developed, including isothermal amplification‐mediated methods, mispairing‐guided polymerase extension, restriction endonuclease‐mediated ligation, in vitro homologous recombination, and redundancy‐minimizing sequence, enabling the construction of tandem repeat tracts spanning kilobases to tens of kilobases in length. In parallel, in vivo construction strategies using model hosts, such as *Escherichia coli* (*E*. *coli*), *Saccharomyces cerevisiae* (*S*. *cerevisiae*), and mammalian cells, are being actively developed to yield longer, more structurally complex, and functionally annotated repeat arrays. Notably, amplification editing (AE) has been reported to achieve duplication of 20 bp to 100 Mb with high efficiency and precision [27]. Here, we review current strategies for constructing repetitive DNA, systematically compare in vitro and in vivo approaches, and discuss the potential of engineered repetitive DNA for the development of genetic manipulation tools (specifically multiplex CRISPR‐Cas system development and artificial chromosome engineering), constructing models of repetitive DNA, and biomaterials synthesis, aiming to guide methodological choice and downstream implementation (Figure 1).

**FIGURE 1:**
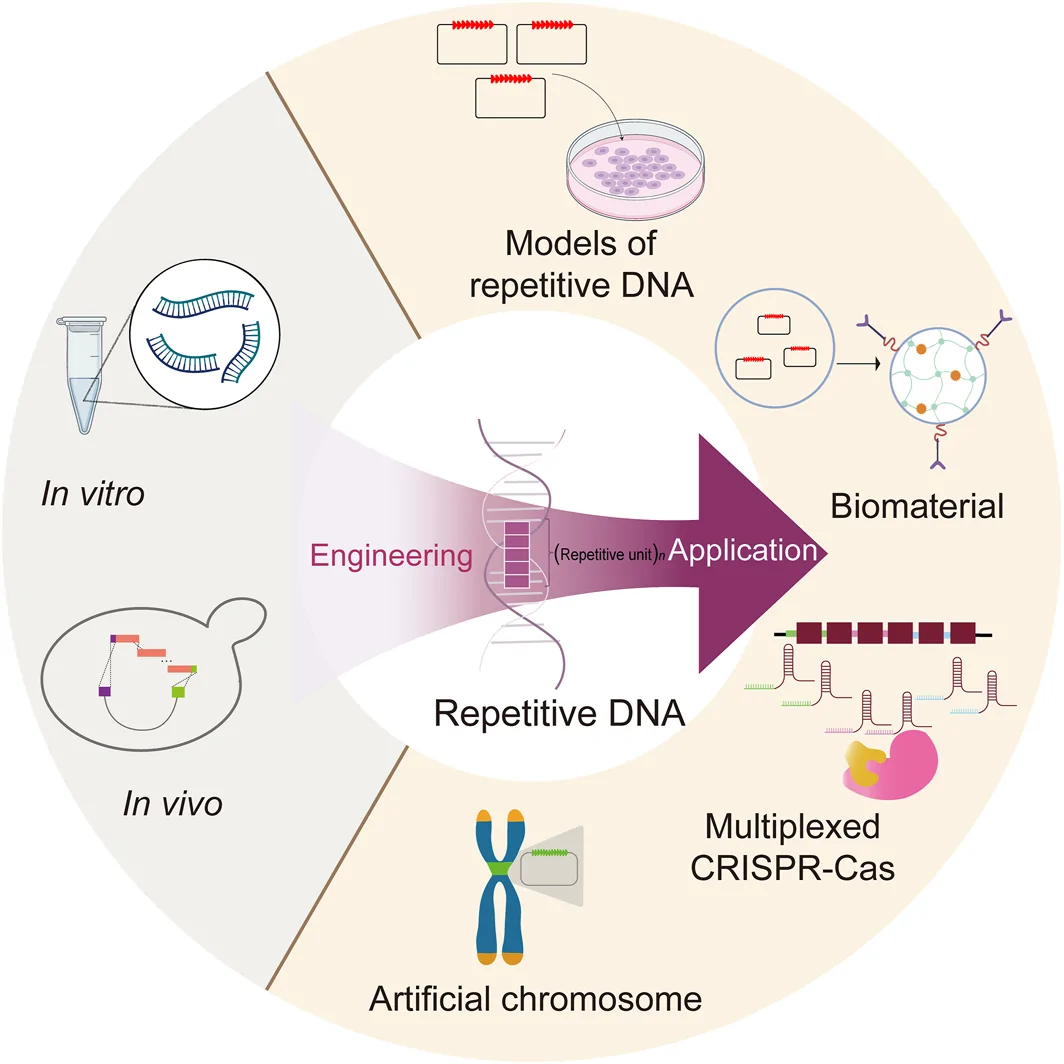
Overview of engineering strategies and applications of repetitive DNA.

## 2 CONSTRUCTION OF REPETITIVE DNA

As the sequencing and annotation of repetitive DNA continue to improve, rapid progress has been made in the construction of repetitive DNA. Among current strategies, in vitro generation of kilobase (kb) to tens‐of‐kb repeat fragments is feasible; however, efficiency declines as fragment length and repeat‐unit homogeneity increase. In vivo *s*trategies are advancing, and in situ construction of chromosome‐scale repetitive arrays in mammalian cells has been enabled by recent research.

### 2.1 In vitro methods for the construction of repetitive DNA

Based on mechanisms of repetitive DNA formation and guiding principles, in vitro strategies for constructing repetitive DNA can be grouped into five broad classes: isothermal amplification‐mediated construction of repetitive DNA, mispairing‐guided polymerase extension for the construction of repetitive DNA, restriction endonuclease‐mediated construction of repetitive DNA, in vitro homologous recombination for the construction of repetitive DNA, and constructing repetitive DNA by reducing sequence redundancy (Figure 2).

**FIGURE 2:**
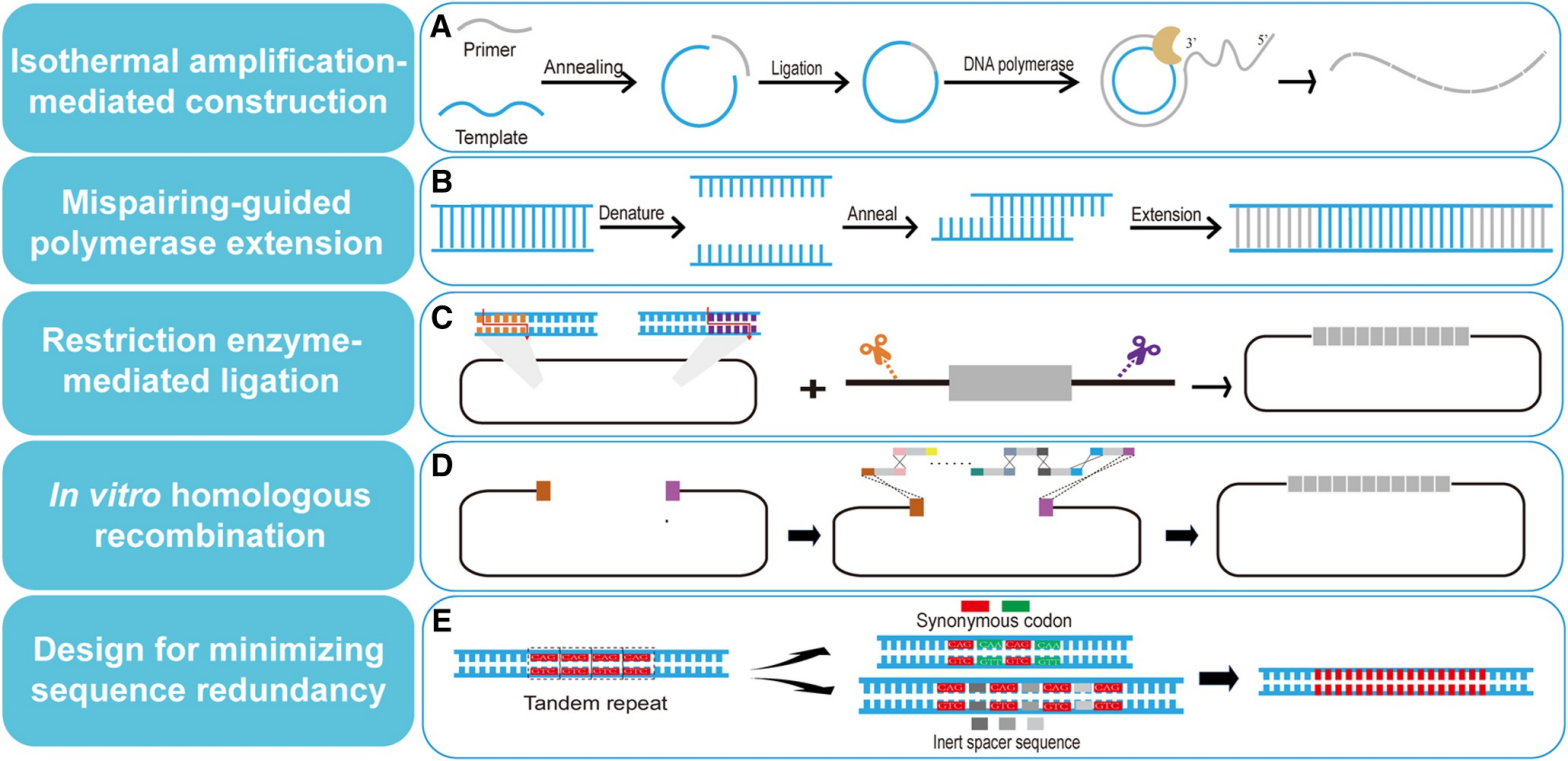
Mechanism‐based in vitro strategies for constructing repetitive DNA. (A) Isothermal amplification‐mediated construction. (B) Mispairing‐guided polymerase extension. (C) Restriction enzyme‐mediated ligation. (D) In vitro homologous recombination. (E) Design for minimizing sequence redundancy.

#### 2.1.1 Isothermal amplification‐mediated construction of repetitive DNA

In highly homogeneous tandem repeats, conventional PCR is prone to strand slippage and template switching, thereby limiting the accurate amplification of long repetitive sequences. Rolling circle amplification (RCA) utilizes circular templates and strand‐displacement DNA polymerases to perform isothermal amplification at around 30°C, producing long single‐stranded DNA (ssDNA) concatemers composed of tandem repeats of the template. Product lengths commonly reach tens of kilobases (around 30–70 kb), depending on the system and conditions [28, 29, 30]. In the absence of additional primers, RCA proceeds with nearly linear accumulation, adding extra primers produces hyperbranched RCA (HRCA) with exponential kinetics [31]. RCA has been widely used to generate simple tandem repeats, functional genetic modules, and scaffolds for DNA nanostructures [32, 33, 34, 35, 36, 37].

In overlap extension rolling circle amplification (OERCA), RCA is combined with overlap‐extension PCR to generate double‐stranded DNA (dsDNA) libraries with tunable repeat copy numbers in a single‐pot reaction. Early studies have shown that OERCA yields a broader, more tunable length distribution than overlap extension PCR (OE‐PCR) or ligation‐based methods during the synthesis of repetitive genes such as elastin‐like polypeptides (ELPs) [38, 39].

Overall, despite these advantages, RCA‐based strategies remain constrained by the need for efficient template circularization, variability in product‐length distributions, and limited portability across sequence contexts and systems. For constructing ultra‐long, high‐identity repeat arrays, these methods are typically combined with complementary strategies and workflows.

#### 2.1.2 Mispairing‐guided polymerase extension for the construction of repetitive DNA

During the replication of highly repetitive DNA, DNA polymerase is prone to strand slippage, a phenomenon characterized by the transient dissociation of strands followed by misaligned reannealing. This phenomenon results in the insertion or deletion of repeat units and is a key mechanism underlying microsatellite locus length variation and in vitro amplification artifacts [40].

The synthesis of long iterative polynucleotide (SLIP) exploits this mechanism: efficient expansion of tandem repeats, such as CAG/CTG, is achieved through repeated cycles of denaturation and annealing, followed by gap filling with a high‐fidelity DNA polymerase. However, the final product length is heterogeneous and not precisely predictable, and cloning and screening are required to isolate constructs of defined length [41, 42, 43]. In enzymatic heat‐cool cycling extension, misaligned double‐stranded intermediates are generated through repeated thermal cycles, producing 5′ overhangs that are extended by DNA polymerase. This enables the synthesis of long repeats from short oligonucleotides, typically yielding products of 0.5–20 kb [44]. For GC‐rich or longer repeat units, broader length distributions are observed, and self‐complementary or concatemeric products are more likely to form [45].

Repeat‐extension PCR (RepEx‐PCR) enables the generation of long tandem repeat arrays from two short complementary oligonucleotides by offset annealing, which creates 5′ or 3′ overhangs. A high‐fidelity DNA polymerase fills 5′ overhangs and, via its 3–5′ proofreading exonuclease, removes 3′ overhangs, producing blunt‐ended products that are progressively extended each cycle to yield kb‐scale arrays. However, blunt‐end ligation is inefficient for long inserts, and long repeats are unstable in *E*. *coli*, alternative hosts may improve cloning and stability [46, 47]. In blocking PCR for DNA repeat expansion (BPRE), an excess partially matching blocking primer anneals to the 5′ portion of the repeat unit, biasing an offset annealing event in which the 3′ overhang of a template primer pairs with the exposed 3′ portion of the repeat. Taq polymerase then extends along the template primer to add one repeat unit per productive cycle, yielding double‐stranded repeat arrays, whereas a subset of molecules fully reanneal and thus skip expansion in a given round [48].

#### 2.1.3 Restriction endonuclease‐mediated construction of repetitive DNA

Standardized, modular, iterative construction strategies integrate genetic elements into an ordered, hierarchical workflow comprising three levels: component, module, and array, allowing parts to be reused across iterations. Under the BioBrick standard, predefined restriction sites in the prefix and suffix enable idempotent assembly. However, this approach introduces sequence scars, and pairwise ligation steps become inefficient for long tandem arrays [49, 50]. Within this framework, quick gene assembly (QGA) has been used in vitro to construct a tandem array of 25 BioBrick units through iterative *Xba* I/*Spe* I digestion and ligation [51]. In Golden Gate assembly, Type IIS restriction enzymes cleave outside their recognition sites to generate custom overhangs, enabling scarless one‐pot, multi‐fragment assembly, which is well suited to constructing ordered repeat arrays [52, 53, 54, 55]. In PS‐Brick, Type IIP and Type IIS enzymes are combined to achieve scarless assembly while preserving part reusability and supporting iterative amplification. However, it is limited by the need to remove internal recognition sites, and its assembly workflow is more complex than that of one‐pot Golden Gate methods [49]. Cloning troublesome repeats in loops (CTRL) utilizes BioBrick or Golden Gate‐based methods to expand arrays twofold or fourfold per cycle, ultimately reaching 256 binding sites, with accuracy and yield gradually declining as length increases [56].

Beyond these modular schemes, recursive ligation‐based approaches have been developed to extend repeat arrays iteratively. In recursive directional ligation (RDL), two restriction enzymes with different recognition sequences are chosen so that their cohesive ends are compatible, enabling iterative, directional ligation to generate repeat arrays. The strategy has been utilized to synthesize libraries of ELPs and to develop seamless cloning workflows for large microsatellite repeat expansions, which are, in principle, applicable to diverse STRs [57, 58]. Although RDL is effective, its utility is limited by sequence constraints imposed by restriction sites embedded within coding regions, substantial background from vector self‐ligation and insert circularization, and the burden of multistep cloning. RDL by plasmid reconstruction (PRe‐RDL) addresses these limitations by reconstructing the plasmid using Type IIS cleavage outside the recognition sites, thereby minimizing background and reducing the retention of restriction sites within the repetitive tract, which improves cloning efficiency and experimental control [59, 60].

#### 2.1.4 In vitro homologous recombination for the construction of repetitive DNA

In overlap‐based in vitro construction, about 20–40 bp overlaps are appended to the ends of fragments, enabling directional, scarless joining via base pairing. Overlaps are readily appended by PCR, providing flexibility in experimental design. The Gibson assembly exemplifies this approach: a 5′ exonuclease creates 3′ ssDNA overhangs that are filled by DNA polymerase and sealed by DNA ligase, allowing multi‐fragment assembly in a single isothermal reaction. Although effective for complex constructs, high‐copy repeats increase mis‐annealing, template switching, and rearrangements, potentially yielding incorrect junctions [61, 62].

To address these limitations, unique nucleotide sequences (UNS) can flank each repetitive module as orthogonal linkers, in conjunction with Gibson or other recombination‐based cloning strategies, ensuring the accurate and ordered formation of repeat arrays [63, 64, 65]. Similarly, modular overlap‐directed assembly with linkers (MODAL) utilizes standardized adapters and orthogonal overlaps to assign unique addresses to modules, improving accuracy, scalability, and modularity in building repeat‐containing constructs [66].

#### 2.1.5 Constructing repetitive DNA by reducing sequence redundancy

The construction of repetitive DNA typically relies on restriction enzyme digestion and homology‐directed scarless assembly. However, under high sequence identity, overlaps are prone to mis‐annealing and cross‐hybridization, incorrect junctions can occur, construct instability increases, and workflows become more complex. To mitigate these challenges, one approach is derepetitization (synonymous recoding), in which the nucleotide sequence is rewritten without altering the encoded protein. For example, the polyglutamine (polyQ) CAG tract can be recoded as a synonymous sequence incorporating CAA interruptions [67]. Computational approaches such as combinatorial codon scrambling [68, 69, 70] and tandem repeat DNA sequence redesign (TReSR) [71] can minimize nucleotide‐level identity between adjacent repeat units, thereby improving the accuracy of amplification.

When codon recoding is not feasible, algorithmic design of non‐repetitive genetic parts, such as nonrepetitive parts calculator (NRPC)‐generated promoter libraries, offers a validated strategy to reduce local sequence identity while maintaining regulatory function [72]. For co‐expression of multiple single‐guide RNAs (sgRNAs), non‐repetitive part libraries can be generated through directed mutagenesis guided by algorithmic constraints, thereby minimizing internal sequence redundancy [73, 74]. Alternatively, a single‐promoter design can transcribe the entire sgRNA array, reducing the need for multiple identical promoter elements [75, 76, 77]. In addition, predefined non‐repetitive linkers placed at cleavage sites enable the one‐pot assembly of multi‐site CRISPR arrays while reducing mis‐annealing caused by repetitive elements [78, 79] (Table 1).

**TABLE 1:** In vitro methods for constructing repetitive DNA.

Mechanism	Method	Maximum repeat sequence size	Reference
Isothermal amplification‐mediated	Rolling circle amplification (RCA)	Approaches 70 kb	[27, 28, 31, 32, 33]
	Overlap extension rolling circle amplification (OERCA)	1.4 kb	[37, 38]
Mispairing‐guided polymerase extension	Synthesis of long iterative polynucleotide (SLIP)	0.57 bp	[41, 42]
	Enzymatic heat‐cool cycling extension	> 20 kb	[43, 44]
	Repeat‐extension PCR (RepEx‐PCR)	0.6 kb (reach tens of kb theoretically)	[45, 46]
	Blocking PCR for DNA repeat expansion (BPRE)	1 kb	[47]
Restriction endonuclease‐mediated	Quick gene assembly (QGA)	1.714 kb	[50]
	Golden Gate	4.8 kb (256 repeat units)	[51, 53, 54, 55]
	PS‐Brick	Up to 3 sgRNA repeats	[48]
	Recursive directional ligation (RDL)	3.6 kb	[56, 57, 58, 59]
In vitro homologous recombination	Gibson	3.384 kb	[60, 61]
	Unique nucleotide sequences (UNS)‐guided assembly	64 kb	[62, 63, 64]
	Modular overlap‐directed assembly with linkers (MODAL)	5–20 kb	[65]
Design for minimizing sequence redundancy	Based on codon degeneracy	2.1 kb	[66, 79]
	Combinatorial codon scrambling	2.52 kb	[67, 68, 69]
	Tandem repeat DNA sequence redesign (TReSR)	0.534 kb	[70]
	Co‐expression of multiple sgRNAs	NA	[53, 74, 76, 77, 78, 80]

### 2.2 In vivo methods for the construction of repetitive DNA

Engineering repetitive DNA commonly relies on in vitro assembly, a laborious process that is difficult to scale to vast constructs. By contrast, approaches based on host cells are more effective for assembling larger DNA constructs in vivo. Although strategies focused on repeats remain limited, in vivo assembly has been actively explored in *E*. *coli*, *S*. *cerevisiae*, and mammalian cells (Figure 3).

**FIGURE 3:**
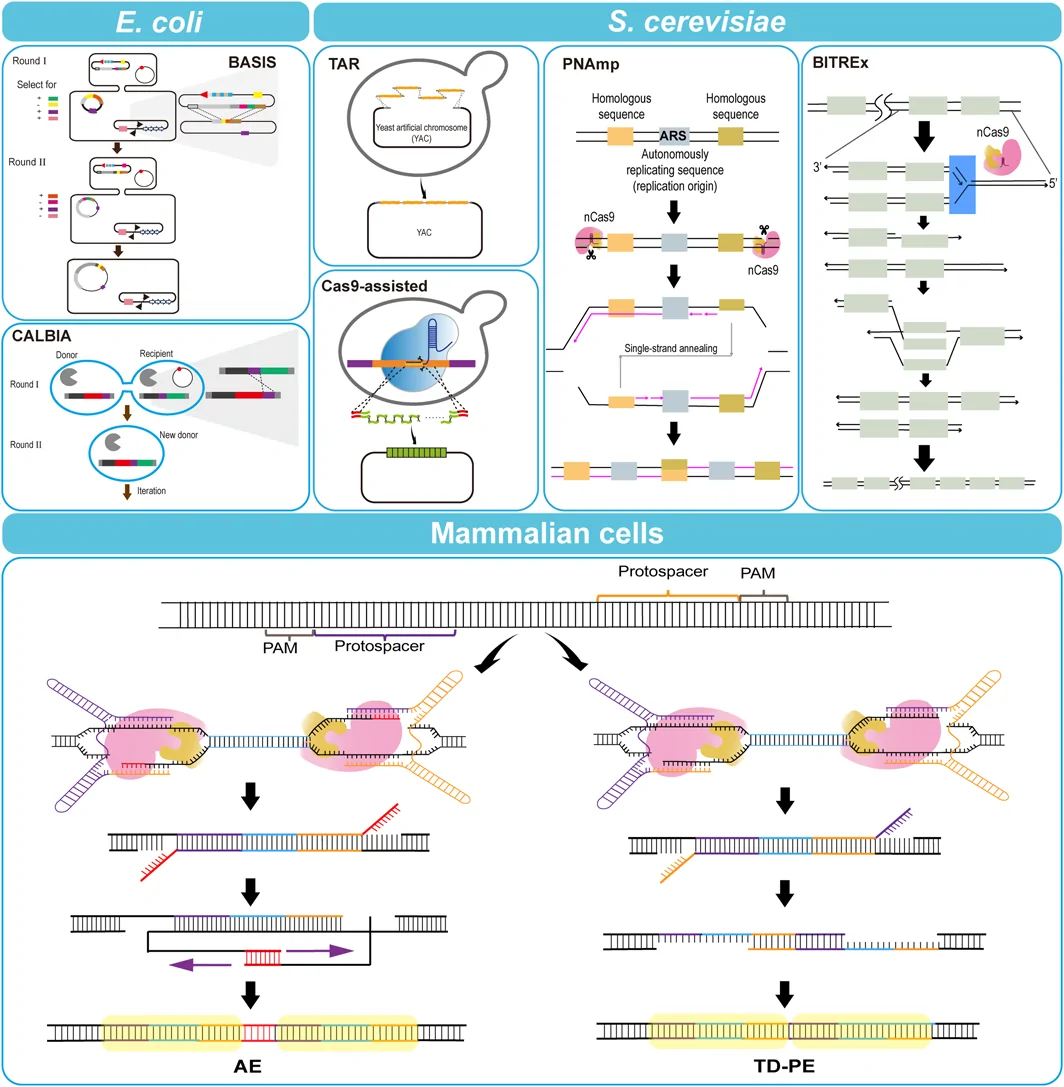
Schematic of in vivo strategies for engineering repetitive DNA across *E. coli*, *S. cerevisiae*, and mammalian cells.

#### 2.2.1 Construction of repetitive DNA in *E*. *coli*

Leveraging its low endogenous homologous recombination, *E*. *coli* provides a stable host for large DNA and can stably maintain human fragments of tens to hundreds of kilobases. However, conventional workflows are not readily scalable or iterative. Recently, bacterial artificial chromosome (BAC) stepwise insertion synthesis (BASIS) [80] and conjugation associated linear‐bacterial artificial chromosome iterative assembling (CALBIA) [81] have demonstrated truly iterative construction in *E*. *coli*, extending human genomic segments to the megabase scale and providing a practical route and methodological framework for programmable in vivo construction of repetitive DNA.

BASIS utilizes a BAC scaffold and alternates Cas9 excision with λ‐Red recombination and conjugative transfer, relying solely on short terminal homology arms to facilitate iterative, scarless joining of large human DNA blocks. This workflow culminated in a 1.1 Mb segment from human chromosome 21 that retained LINE/SINE elements, G‐quadruplex motifs, and STRs, such sequences are comparatively unstable in *S*. *cerevisiae*, making *E*. *coli* a more suitable host for carrying and amplifying repeat‐rich human DNA [80].

CALBIA, by contrast, builds linear BACs and exploits host RecA‐mediated single crossover homologous recombination during bacterial conjugation to achieve day‐scale iterations, joining fragments in the hundreds of kb range without the use of Cas9 or λ‐Red, thereby reducing the risk of mis‐recombination at small repeat tracts after Cas9 cleavage. In practice, CALBIA has been used to construct a 1.07 Mb IgHV cluster and about 1.1–1.2 Mb segments from 18p11.2 in *E*. *coli*. Free linear BACs carrying near Mb inserts are deletion‐prone in AT‐rich or repeat‐dense regions, whereas linear chromosome integration confers long‐term genetic stability [81].

#### 2.2.2 Construction of repetitive DNA in *S*. *cerevisiae*

*S*. *cerevisiae* is widely used for in vivo DNA construction owing to its highly efficient homologous recombination, mature genetic toolkits, and capacity to maintain large DNA constructs. Developing more versatile strategies for generating synthetic repeat arrays within the *S*. *cerevisiae* genome would benefit a broad range of synthetic biology applications.

Transformation‐associated recombination (TAR) combined with RCA enables the rapid construction of tandem repeat arrays. Following an initial in vitro RCA step, short repeat units are first concatemerized into multi‐kb products, and a single TAR step then assembles these into arrays of tens of kilobases. Additional cycles can extend the arrays to the hundreds‐of‐kilobases range. However, single‐round TAR yields of long arrays are low, and the workflow is relatively complex and time‐consuming [82, 83]. Alternatively, CRISPR with *S*. *cerevisiae* homologous recombination generates targeted double‐strand breaks (DSBs), followed by cotransformation of partially overlapping fragments, enabling direct in vivo generation of approximately 1–100 copies. The approach is helpful for medium copy numbers and complementary to in vitro preassembly, but it does not allow the exact number of repeats to be predetermined [84].

Paired nicking‐induced amplification (PNAmp) places a pair of Cas9 nickases (nCas9) on opposite strands flanking a replication origin at the boundaries of the target segment so that the two forks emanating from that origin collapse at the Nick sites, generating paired one‐ended DSB. After end processing, single‐strand annealing (SSA) between co‐oriented direct repeats joins the intervening segment head‐to‐tail, producing a segmental duplication that can be iteratively expanded. This approach induces duplication of segments as large as 1 Mb with efficiencies exceeding 10% in the budding yeast [85]. Building on this foundation, BIR‐mediated tandem repeat expansion (BITREx) likewise couples site‐specific nicking to replication‐dependent repair, instead invoking break‐induced replication (BIR) from an upstream repeat unit to drive head‐to‐tail copy number gains. However, because this process is replication‐coupled, it does not apply to nondividing cells [86].

Beyond repeat‐specific strategies, a segmented, iterative scheme combining long homology arms, cleavage mediated by Cas9, and TAR synthesis resulted in a human AZFa segment of 1.14 Mb with about 70% repetitive content [87]. Construction of a repetitive array by transformation (CAT) introduces linear fragments with flanking homology in a single step, endogenous recombination with in situ tandem amplification yielded ribosomal DNA (rDNA) arrays with about 10–15 copies [88]. The yeast life cycle (YLC) assembly leveraged the YLC to iteratively build a human IGH region of 1.26 Mb, rich in repeats. However, the build remained unstable, and dispersed repeats caused localized deletions [89].

#### 2.2.3 Construction of repetitive DNA in mammalian cells

Because homology‐directed repair (HDR) is relatively inefficient for joining multi‐fragment sequences in mammalian cells, the direct construction of repeat‐rich constructs remains challenging. To build tandem repeats directly in situ, prime editing‐based strategies now enable programmable duplication of sequences. Tandem duplication via prime editing (TD‐PE) utilizes paired pegRNAs to program precise duplication segments ranging from 50 bp to 10 kb segments in mammalian cells [90]. AE couples nCas9‐reverse transcriptase with paired PAM‐out pegRNAs to trigger templated DNA amplification, supporting duplications from tens of base pairs to chromosomal scales without the need for donor DNA [27]. However, these methods mainly produce in situ tandem repeats for pre‐existing sequences in the genome, which makes it difficult to amplify exogenous or synthetic sequences. In addition, yeast origin strategies have been adapted for use in mammals, BITREx utilizes BIR from nickase‐induced fork breakage to expand tandem arrays in mammalian cells, providing complementary routes to engineer repetitive DNA [86] (Table 2).

**TABLE 2:** In vivo methods for constructing repetitive DNA.

In vivo host	Method	Maximum repeat sequence size	References
*Escherichia coli*	Bacterial artificial chromosome stepwise insertion synthesis (BASIS)	1.1 Mb	[79]
	Conjugation associated linear‐bacterial artificial chromosome iterative assembling (CALBIA)	1.07 Mb	[80]
*Saccharomyces cerevisiae*	Transformation‐associated recombination (TAR)	1 Mb	[81, 82, 85]
	Cas9‐assisted homologous recombination assembly	Up to 16.5 kb (100 repeats)	[83]
	Paired nicking‐induced amplification (PNAmp)	About 1 Mb	[84]
	BIR‐mediated tandem repeat expansion (BITREx)	About 1 Mb	[85]
Mammalian cells	Tandem duplication via prime editing (TD‐PE)	10 kb	[91]
	Amplification editing (AE)	100 Mb	[26]

## 3 APPLICATIONS OF ENGINEERING REPETITIVE DNA

### 3.1 Development of genetic manipulation tools

Repetitive DNA engineering is catalyzing the development of next‐generation genetic manipulation tools. Multiplex CRISPR‐Cas architectures utilize modular repeat arrays to coordinate editing or regulation at multiple loci, whereas artificial chromosome engineering employs precisely constructed centromeric repeats to nucleate the centromere and kinetochore assembly, thereby promoting mitotic stability. Together, these capabilities translate sequence programmability into scalable genomic control, expanding the practical reach of genetic manipulation.

Multiplex CRISPR technology allows for the precise modification of multiple genomic targets within a single experimental setup by integrating multiple guide RNA sequences into a single expression system and pairing them with the corresponding Cas proteins [92]. However, sequence similarity among units promotes mis‐annealing, recombination, and misjoining. To address this, non‐repetitive array design strategies, together with modular cloning frameworks, are commonly used to ensure stable construct assembly and efficient expression. These advances have broadened the application of multiplex CRISPR across diverse systems, including microbes, mammals, and plants.

Multiplex genome editing is typically achieved using multiple sgRNAs, often organized as sgRNA arrays, to target multiple loci in parallel and generate indels or other editing outcomes [75, 78, 91, 93, 94, 95, 96, 97, 98]. Multi‐sgRNA arrays enable chromosome‐scale engineering (kb to Mb), including deletions, inversions, translocations, and reduction or near elimination of entire chromosomes, providing tools to probe genome architecture and model chromosomal disorders [99]. For transcriptional control, dCas effectors enable the simultaneous targeting of multiple genes via CRISPR interference (CRISPRi) and CRISPR activation (CRISPRa), facilitating pathway rewiring and coordinated upregulation [74, 77, 100, 101, 102, 103]. Multiplex base editing uses gBlock or Golden Gate assembled sgRNA cassettes with cytidine or adenine base editors (CBEs/ABEs) to modify dozens of sites in a single transfection, including broad TAG to TAA codon replacement [104].

The de novo construction of artificial chromosomes requires the precise synthesis and efficient assembly of large‐scale repetitive DNA arrays to rebuild essential functional domains that nucleate kinetochores, support replication, and ensure stable segregation. In human systems, a synthetic chromosome 17 alpha‐satellite higher‐order repeat (HOR) unit of 2.7 kb has been directionally multimerized into about 86 kb tandem arrays and cloned into BAC‐based vectors. Upon transfection into HT1080 cells, these BAC constructs can seed de novo centromeres and give rise to mitotically stable human artificial chromosomes (HACs) [105]. An alternative pipeline couples RCA in vitro with TAR in yeast to generate about 120–140 kb alphoid DNA arrays. Operator sequences, such as TetO or LacO, can be embedded to permit TetR/LacI tethering for the visualization and manipulation of HAC chromatin and gene expression. De novo HACs formed from these arrays can further expand to megabase scale and exhibit long‐term mitotic stability [82, 106, 107].

In plant systems, plant artificial chromosomes can also be constructed de novo by combining the synthesis of repetitive arrays with targeted recruitment of kinetochore proteins. For example, in *Arabidopsis thaliana*, a LacI‐CENH3 fusion protein has been used to nucleate neokinetochores at previously inserted LacO tandem repeats, generating mitotically unstable dicentric chromosomes and providing a proof‐of‐concept route toward artificial chromosomes in plants [108, 109]. Similarly, in maize, the synthesis of an ABS4 array containing approximately 2400 repeat units, followed by LexA–CENH3‐mediated targeting, has resulted in the formation of plant artificial chromosomes that are stably transmitted through meiosis [110, 111].

### 3.2 Constructing models of repetitive DNA

More than 60 repeat expansion diseases, most of which primarily affect the nervous system, have been identified to date [7, 8, 112]. Accordingly, engineering in vitro models of repeat expansion diseases is critical for mechanistic dissection, quantitative phenotyping, and scalable testing of candidate interventions.

To address this need, multiple strategies have been employed to reconstruct pathogenic repetitive DNA with precision. For example, restriction enzyme ligation combined with RCA produces CTG arrays at the kb scale, and plasmids bearing hundreds to thousands of CTG repeats reproduce RNA toxicity phenotypes in human cells [113]. SLIP readily yields defined ATXN3 CAG/CAA variants for expression in neuronal models, allowing for the dissection of RNA versus protein toxicity [42]. Additionally, expansion of HTT CAG repeats to approximately 114 units was enabled by Golden Gate cloning combined with CRISPR/Cas9‐mediated HDR, followed by site‐specific integration into the human genome, yielding isogenic cell lines with varying repeat lengths for Huntington’s disease research [114]. In *Drosophila* models, RDL was used to construct and express vectors containing 3, 36, or 103 pure GGGGCC repeat units, as well as “RNA‐only” constructs in which 6‐bp stop codon‐containing interruptions were inserted every 12 repeats, thereby enabling in vivo modeling of C9ALS/FTD [115]. AE and TD‐PE have enabled the precise construction of disease‐relevant repeats at multiple loci in cell and stem‐cell contexts, and are emerging as valuable platforms for biological research and disease modeling [27, 90].

Beyond human and animal disease models, engineered repetitive DNA has also been utilized to investigate chromatin regulation in plants and to examine rDNA array dynamics in yeast. Depositing CEN180 arrays into introns nucleates intragenic heterochromatin, marked by CG/CHG/CHH DNA methylation and H3K9me2, in a repeat‐dosage–dependent manner. The histone H3K9me2 demethylase IBM1 normally restricts outward spreading, whereas its loss allows this heterochromatic state to be heritably propagated [116]. Inverted repeat RNAs targeting FLOWERING LOCUS T (FT) introns activate intragenic RNA‐directed DNA methylation (RdDM) to upregulate FT while preserving its diurnal/spatiotemporal expression pattern [117]. Moreover, tandem booster1 (b1) repeats drive trans‐allelic paramutation, which is MOP1‐dependent and associated with small interfering RNAs (siRNAs), resulting in transgenerational silencing [118]. Focusing on rDNA, yeast synXII enables the deletion and regeneration of the multigene rDNA array at distinct chromosomal loci and ITS swapping [119], providing a programmable model for nucleolar organization, array copy number homeostasis, and the functional constraints of rRNA processing.

### 3.3 Biomaterial synthesis via programmable repeat construction

Biological materials are central to medicine, environmental protection, and industrial manufacturing, and a clear trend toward biodegradability, programmability, and high biocompatibility has been observed. Among fabrication strategies, repetitive sequence construction is used as a key strategy to translate molecular design into functional material performance [120].

In protein materials, recombinant repetitive protein polymers, such as ELPs, amyloid‐spider silk proteins, squid ring‐teeth proteins, and resilin‐like polypeptides (RLPs), have made significant advancements. Using RDL, researchers have expressed ELPs from genes encoding up to about 240 VPGXG repeats [57] and have cloned recombinant spider silk genes of defined length [121]. Golden Gate assembly has been successfully used to construct genes for amyloid‐spider silk proteins, ELPs, and squid ring‐teeth proteins [122, 123, 124]. OERCA enables one‐pot, high‐throughput synthesis of repetitive genes and has been used to demonstrate *E*. *coli* expression of ELP libraries and protease‐responsive GLP‐1 protein polymers [39]. The codon scrambling algorithm was successful in synthesizing a wide range of repeated protein polymers, including ELPs, RLPs, and collagen‐like polypeptides, with repeat numbers of up to 150 [68]. Collectively, these methods enable the precise programming of chain length and motif composition in repetitive protein polymers, thereby allowing for tunable thermoresponsive phase transitions and other material functions that underpin controlled drug release and tissue engineering applications [57, 62, 120, 125, 126, 127].

In nucleic acid nanomaterials, RCA continuously produces long ssDNA concatemers with tandem repeat units under isothermal conditions. These RCA products can be processed via metal coordination, crosslinking, or mineralization to build DNA nanoflowers (DNFs) [128] and DNA hydrogels [33, 129, 130]. Incorporating chemically modified deoxyribonucleoside triphosphates (dNTPs) enables the direct encoding of fluorescent moieties, ligands, or therapeutics into the structures, allowing for drug and gene delivery, imaging, biosensing, and the creation of degradable scaffolds. These strategies transform programmability at the molecular level of repetitive sequences into manufacturability and functional diversification.

## 4 CONCLUSIONS AND PERSPECTIVES

In the era of complete genomes, moving from “reading” to “writing” is not only a natural extension of sequencing but also a necessary test of many genomic inferences. Nevertheless, the precise construction of long repeats remains constrained by both chemistry and biology: on the chemical side, tract length and secondary‐structure formation depress synthesis and joining efficiencies [131]; on the biological side, highly homologous repeats are intrinsically unstable during cloning and propagation, leading to copy‐number loss and rearrangements [132, 133]. In vitro strategies can generate tens of kb repeat tracts under controlled conditions, but their limitations are broadly predictable. Restriction endonuclease‐based routes suffer from the short overhangs produced by many enzymes, which afford insufficient specificity in repeat‐rich contexts and complicate iterative joining. Restriction endonuclease‐independent methods also face elevated mis‐annealing and chimera risks in high‐repeat, GC‐rich, or G‐quadruplex‐forming sequences, and they remain labor‐intensive and length‐limited. Once constructs exceed about 10–20 kb, shear sensitivity and reduced transformability often translate into host incompatibility or instability [134, 135].

By contrast, in vivo approaches offer clear advantages in achievable length. De novo reconstruction of Mb‐scale repeat‐rich segments is feasible in yeast [87]. However, the repetitive nature of many human genomic regions can render them unstable in that host [89], underscoring the need to tune recombination pathways and repair balance to stabilize complex architectures. An alternative is to build in hosts with lower endogenous homologous recombination, such as *E*. *coli* or mammalian cells. In *E*. *coli*, low natural recombination supports the maintenance of human DNA fragments on the order of hundreds of kilobases. Methods such as BASIS and CALBIA use iterative BAC/linear‐BAC workflows to enable programmable in vivo construction of repeat‐rich DNA at near‐megabase scales, offering a practical route and design playbook [80, 81]. In mammalian cells, tandem duplication via TD‐PE and AE can install precise in situ duplications with high efficiency [27, 90]. However, they act on pre‐existing genomic sequences; consequently, merging them with synthetic genomics would generalize the approach to the de novo, programmable installation of large‐scale synthetic DNA replication.

Overall, most current research primarily circumvents the intrinsic instability of repetitive DNA. Building on existing insights into repetitive sequence stability from molecular dynamics, structural biology, and epigenetics, the next stage should shift toward actively regulating the stability of repetitive DNA. At the host strain, a possible direction is to modulate homologous recombination capacity. This could involve reducing the expression of recombination proteins, constructing a recombination‐deficient strain, or selecting naturally low‐recombination hosts to enhance the ability of cells to stably maintain repetitive DNA, thereby increasing the attainable length and complexity of engineered repeat constructs. A complementary approach is to modulate the accessibility of repetitive sequences by harnessing epigenetic pathways. Targeted recruitment of heterochromatin‐forming factors to repeat domains could lower local accessibility, suppress recombination, and establish boundary‐based chromatin compaction as a tunable layer for stabilizing repetitive DNA. In parallel, these strategies can be complemented by advances in computational biology and AI‐guided design. Computational and AI‐assisted approaches can identify high‐risk repetitive segments, generate low‐repetition building blocks for redesigning repeat units, and rewrite unstable regions while preserving the encoded protein structure or regulatory function, thereby facilitating the construction of longer and more stable repetitive DNA arrays. Within this framework, a shift from “difficult to synthesize” toward “precisely controllable” could improve the manufacturability and predictability of complex repeat constructs, providing a firmer methodological footing for higher‐order artificial genomes and synthetic life systems.

Advances in repetitive DNA engineering are opening opportunities across diverse domains. In cellular and animal disease models, these methods are shaping a standardized, reproducible framework for mechanistic studies and high‐throughput screening. In parallel, they hold promise for converting the molecular programmability of repetitive DNA into manufacturability and functional diversification. When coupled with heterologous expression, they also provide practical routes to the synthesis of high‐value biomaterials. At the same time, precise, scalable repeat‐rich constructs are a prerequisite for multiplex CRISPR and artificial chromosome engineering. Looking ahead, sequencing and synthesis are likely to continue converging: the costs of building and testing chromosome‐scale DNA are expected to decline, whereas methods for engineering repetitive DNA can be anticipated to improve in fidelity, scale, and predictability, thereby accelerating the translation from principles to functional sequences and systems.

## AUTHOR CONTRIBUTIONS

**Shuting Ma:** Conceptualization; investigation; visualization; writing—original draft preparation; writing—review and editing. **Yali Cui:** Investigation; writing—original draft preparation. **Yi Wu:** Conceptualization; project administration; supervision; writing—original draft preparation; writing—review and editing.

## CONFLICT OF INTEREST STATEMENT

The authors declare no conflicts of interest.

## ETHICS STATEMENT

This article does not contain any studies with human participants or animals performed by any of the authors.

## Data Availability

Data sharing is not applicable to this article as no datasets were generated or analyzed during the current study.
